# Camera settings and biome influence the accuracy of citizen science approaches to camera trap image classification

**DOI:** 10.1002/ece3.6722

**Published:** 2020-10-06

**Authors:** Nicole Egna, David O'Connor, Jenna Stacy‐Dawes, Mathias W. Tobler, Nicholas Pilfold, Kristin Neilson, Brooke Simmons, Elizabeth Oneita Davis, Mark Bowler, Julian Fennessy, Jenny Anne Glikman, Lexson Larpei, Jesus Lekalgitele, Ruth Lekupanai, Johnson Lekushan, Lekuran Lemingani, Joseph Lemirgishan, Daniel Lenaipa, Jonathan Lenyakopiro, Ranis Lenalakiti Lesipiti, Masenge Lororua, Arthur Muneza, Sebastian Rabhayo, Symon Masiaine Ole Ranah, Kirstie Ruppert, Megan Owen

**Affiliations:** ^1^ Duke University Nicholas School for the Environment Durham NC USA; ^2^ San Diego Zoo Institute for Conservation Research Escondido CA USA; ^3^ Save Giraffe Now Dallas TX USA; ^4^ The Faculty of Biological Sciences Goethe University Frankfurt am Main Germany; ^5^ Department of Physics Lancaster University Lancaster UK; ^6^ Science and Technology University of Suffolk Ipswich UK; ^7^ Giraffe Conservation Foundation Windhoek Namibia; ^8^ Instituto de Estudios Sociales Avanzados (IESA‐CSIC) Cordoba Spain; ^9^ Loisaba Conservancy Nanyuki Kenya; ^10^ Namunyak Wildlife Conservation Trust Archer's Post Kenya

**Keywords:** amazon, crowdsource, image processing, kenya, serengeti, trail camera, volunteer

## Abstract

Scientists are increasingly using volunteer efforts of citizen scientists to classify images captured by motion‐activated trail cameras. The rising popularity of citizen science reflects its potential to engage the public in conservation science and accelerate processing of the large volume of images generated by trail cameras. While image classification accuracy by citizen scientists can vary across species, the influence of other factors on accuracy is poorly understood. Inaccuracy diminishes the value of citizen science derived data and prompts the need for specific best‐practice protocols to decrease error. We compare the accuracy between three programs that use crowdsourced citizen scientists to process images online: Snapshot Serengeti, Wildwatch Kenya, and AmazonCam Tambopata. We hypothesized that habitat type and camera settings would influence accuracy. To evaluate these factors, each photograph was circulated to multiple volunteers. All volunteer classifications were aggregated to a single best answer for each photograph using a plurality algorithm. Subsequently, a subset of these images underwent expert review and were compared to the citizen scientist results. Classification errors were categorized by the nature of the error (e.g., false species or false empty), and reason for the false classification (e.g., misidentification). Our results show that Snapshot Serengeti had the highest accuracy (97.9%), followed by AmazonCam Tambopata (93.5%), then Wildwatch Kenya (83.4%). Error type was influenced by habitat, with false empty images more prevalent in open‐grassy habitat (27%) compared to woodlands (10%). For medium to large animal surveys across all habitat types, our results suggest that to significantly improve accuracy in crowdsourced projects, researchers should use a trail camera set up protocol with a burst of three consecutive photographs, a short field of view, and determine camera sensitivity settings based on in situ testing. Accuracy level comparisons such as this study can improve reliability of future citizen science projects, and subsequently encourage the increased use of such data.

## Graphical abstract

We show that the accuracy levels of trail‐camera image classification by citizen scientists are affected by habitat type and trail‐camera set up. By comparing the accuracy results from three camera trap citizen science projects, we found that setting trail cameras to capture 3 images per burst, testing the appropriate camera sensitivity, and a shorter field of view resulting from dense vegetation may significantly improve citizen scientist image classification accuracy when compared to classifications by experts.

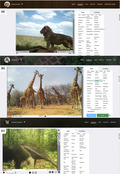

## 1 INTRODUCTION

Citizen science, the practice of volunteer participation in scientific research, has long played a role in the collection and analysis of data, and has provided public access to scientific information and education. Evidence of early examples date back to the late nineteenth century where North American lighthouse keepers began collecting bird strike data and volunteer‐based bird surveys began in Europe (Dickinson, Bonney, & Fitzpatrick, 2015). Beginning in 1900, the National Audubon Society's annual Christmas Bird Count is still active over a century later, and recently documented that net bird populations in the United States have declined by three billion individuals over the past 50 years (Dickinson et al., 2015; Rosenberg et al., 2019). It is clear that science has benefitted from the use of volunteers as a cost‐saving, and in some cases, more rapid and broad scale means of data collection and processing (Tulloch, Possingham, Joseph, Szabo, & Martin, 2013). Additionally, engaging citizen scientists increases scientific literacy among the public and spreads awareness about research (Jordan, Gray, Howe, Brooks, & Ehrenfeld, 2011; Mitchell et al., 2017).

With recent technological advancements, the availability and diversity of projects suitable for public participation have increased dramatically (Dickinson, Zuckerberg, & Bonter, 2010; Silvertown, 2009; Tulloch et al., 2013). Online citizen science research projects have been developed for a range of species and programs around the world, for example, observing fireflies (Firefly Watch), mapping herpetological observations (HerpMapper), and identifying roadside wildlife (Wildlife Road Watch) (Swanson, Kosmala, Lintott, & Packer, 2016). Despite some skepticism about using data produced by nonexperts (Dickinson et al., 2010; Foster‐Smith & Evans, 2003), numerous studies have shown that citizen science can produce accurate results for ecological science (Kosmala, Wiggins, Swanson, & Simmons, 2016; Sauermann & Franzoni, 2015).

A common and increasing use for citizen science in ecological studies is for the placement and collection of motion‐activated cameras, as well as the extraction and analysis of the resulting wildlife images. Motion‐activated cameras (hereafter “camera traps”) have revolutionized wildlife science, providing a robust and noninvasive mode for ecological data collection on a wide range of species (O'Connell, Nichols, & Karanth, 2010). Camera traps are being used to gather data on species’ population sizes and distributions, habitat use, and behavior, thereby facilitating better understanding and protection of natural ecosystems (Agha et al., 2018; McShea, Forrester, Costello, He, & Kays, 2016; Moo, Froese, & Gray, 2018; O'Connor et al., 2019). Camera traps are also extremely useful for capturing rare or elusive species (Pilfold et al., 2019; Tobler, Carrillo‐Percastegui, Pitman, Mares, & Powell, 2008) and discovering new species all together (Rovero & Zimmermann, 2016). A disadvantage of camera traps is the significant time and resource commitment needed to support the review and classification of images, resulting in cases where data are left unanalyzed (Jones et al., 2018; Norouzzadeh et al., 2018). Tabak et al. (2018) estimated that a person can process approximately 200 camera trap images per hour, a rate that slows with fatigue. In the case of Wildwatch Kenya, a grid of camera traps placed throughout two conservancies in Northern Kenya collected over 2 million images in the three years of deployment (J. Stacy‐Dawes, personal comment, January 2020). At the rate of 200 images/hour, assuming a typical 40‐hr work week, it would take a single researcher 4.8 years (1,250 days) to complete sorting and classifying this dataset of images.

A variety of approaches have been used to process large camera trap datasets including expert processing, trained volunteers, untrained volunteers, and automated processing using computer vision and machine learning (Table 1), each with benefits and drawbacks (Ellwood, Crimmins, & Miller‐Rushing, 2017; Jordan et al., 2011; Kosmala et al., 2016; Mitchell et al., 2017; Norouzzadeh et al., 2018; Silvertown, 2009; Swanson et al., 2016; Tabak et al., 2018; Torney et al., 2019; Tulloch et al., 2013; Willi et al., 2019). Crowdsourcing, the process of outsourcing a task to a large number of people, generally through an online platform, has become a new approach to citizen science. Numerous publications suggest that multiple nonexpert volunteers can be as accurate as a single expert for tasks such as reviewing camera trap images, aerial survey images, and astronomic imagery (Spielman, 2014; Swanson et al., 2016; Torney et al., 2019). This “wisdom of crowds” allows outsourcing of analytical tasks to nonexpert volunteers by aggregating responses to produce accurate, usable, and meaningful data products (Kosmala et al., 2016; Swanson et al., 2016; Tulloch et al., 2013).

**TABLE 1:** Camera trap image classification comparison, where “expert” classifications refer to one professional with extensive background or training in wildlife identification (Swanson et al., 2016), “volunteer” classifications are nonexpert citizen scientists that have undergone training (Tulloch et al., 2013), “crowdsourced” classifications are multiple, aggregated volunteer answers combined to obtain one best answer (Swanson et al., 2016), and “automated” classifications utilize machine learning algorithms to automatically identify species within images (Willi et al., 2019)

	Expert	Trained volunteer	Crowdsourced	Automated
Pros	High accuracy and precision Trusted by the scientific community	Cost‐effective^b^ Time‐efficient^b^ Increases scientific and environmental literacy among the public^c^, ^d^ Spreads awareness about the objectives of a research project^b^, ^c^, ^d^	Accuracy comparable to single expert answers^b^, ^f^, ^g^, ^h^ Cost‐effective^b^ Time‐efficient^b^ Increases scientific and environmental literacy among the public^c^, ^d^ Spreads awareness about the objectives of a research project^b^, ^c^, ^d^ Technological advancements are increasing availability and utility^b^, ^j^	Cost‐effective^k^ Most time‐efficient^k^ Substantially reduce human effort^k^ Evidence of high accuracy^a^, ^k^
Cons	Significant time commitment^a^ Significant financial commitment^a^ Single experts are subject to fatigue with time^l^ Often results in unanalyzed data^a^	Subject to error^g^ Distrust by scientific community^h^ Efficiency is poorly understood by the scientific community^e^ Limited by shortage of volunteers^k^ Requires effort to manage and engage the volunteers^g^	Subject to error Distrust by scientific community^h^ Efficiency is poorly understood by the scientific community^e^ Often requires utilization of external platforms^g^ Limited by shortage of volunteers^k^ and inability of smaller projects to recruit a large volunteer base^a^	Large sets of labeled training data required^k^ Accuracy possibly overestimated when applying existing models to new datasets^k^ Novel technology Requires domain knowledge^k^ Low accuracy for rare species^k^

^a^ Norouzzadeh et al. (2018).

^b^ Tulloch et al. (2013).

^c^ Jordan et al. (2011).

^d^ Mitchell et al. (2017).

^e^ Ellwood et al. (2017).

^f^ Torney et al. (2019).

^g^ Swanson et al. (2016).

^h^ Kosmala et al. (2016).

^i^ Silvertown (2009).

^j^ Dickinson et al. (2010).

^k^ Willi et al. (2019).

^l^ Tabak et al. (2018).

While there are published examples documenting accurate analysis of outputs from citizen science camera trap projects (Swanson et al., 2016), there is a deficiency of evidence‐based and standardized best‐practice camera‐trapping protocols that would maximize nonexpert image classification accuracy and species detectability. Given the prominence and scale of camera trap usage, volume of image generation, and utility of using citizen science approaches, there is a clear and pressing need to standardize camera trap protocols in order to maximize citizen scientist accuracy. Meeting this need would increase the use and acceptance of citizen scientists as a reliable approach to monitoring biodiversity trends (Steenweg et al., 2016), among other applications.

This paper aims to provide insight on protocols to increase data quality and reliability from citizen scientist classification of camera trap images. Here, we analyze how habitat type and camera trap settings, including sensor sensitivity and images per burst, influence nonexpert accuracy. We compare the accuracy of three citizen science camera trap projects: Snapshot Serengeti (SS), Wildwatch Kenya (WWK), and Amazoncam Tambopata (ACT) in order to designate best‐practice methods in camera trap protocols to improve citizen scientist accuracy.

## 2 MATERIALS AND METHODS

### 2.1 The Zooniverse Interface

**FIGURE 1:**
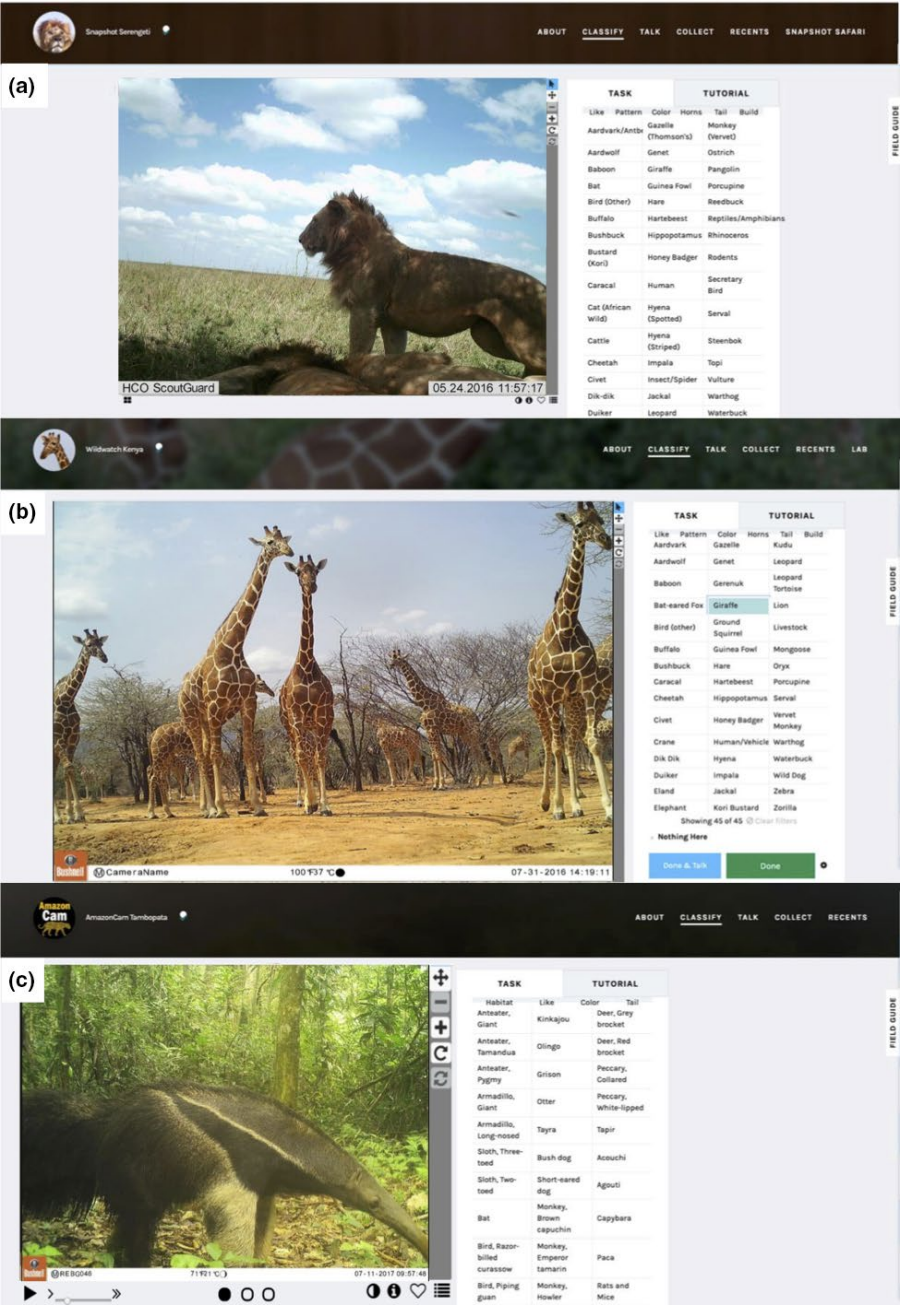
a–c show Zooniverse interfaces of Snapshot Serengeti, Wildwatch Kenya, and AmazonCam Tambopata, respectively. Users classify images by clicking on the appropriate species from the list and selecting the appropriate physical attribute filters

Zooniverse (www.zooniverse.org) is an online citizen science interface that promotes volunteer involvement as a crowdsourcing method for data processing (Cox et al., 2015). Zooniverse users can range in age and expertise (Raddick et al., 2010). The prompts and tutorials set up by each project are meant to successfully guide even the most inexperienced users through the classification process. In the case of the three Zooniverse projects discussed here, volunteers classify species, number of individuals, whether there are young present and (for SS and WWK only) the behavior exhibited for each photograph that appears on the screen. There are guides (Figure 1a–c) to help users identify the species. Volunteers can also classify images that do not contain any animals (i.e., an “empty” image). Each Zooniverse project can customize their retirement rules. For example, after each image is circulated to multiple volunteers, the image will retire after meeting the criteria determined by the project, for example, the first five of classifications are “nothing here,” there are >five nonconsecutive classifications of “nothing here,” there are five matching classifications of a certain species, or there are 10 total classifications without any consensus on a species.

### 2.2 Snapshot Serengeti

Snapshot Serengeti hosts images collected from a camera trap study conducted in the Serengeti National Park, Northern Tanzania (~1.5 million hectares) in order to evaluate spatial and temporal interspecies dynamics (Swanson et al., 2015). This area consists of mostly savanna grasslands and woodlands habitat. A total of 225 Scoutguard (SG565) camera traps were set out across a 1,125 km2 grid, offering systematic coverage of the entire study area. 1.2 million image sets were collected between June 2010 and May 2013 (Swanson et al., 2015). The cameras were set to capture either one or three (majority three) images per burst and were set to “low” sensitivity to minimize misfires due to vegetation (Swanson et al., 2015). On www.snapshotserengeti.org, each camera trap photograph was viewed and classified by 11–57 volunteers (mean = 26) before it was retired (Swanson et al., 2016). This large range resulted from the SS volunteers classifying images faster than they were being collected (Swanson et al., 2016). SS accrued over 28,000 volunteers, who completed the classification of all 1.2 million images collected as of May 2013; however, this project is ongoing.

### 2.3 Wildwatch Kenya

Wildwatch Kenya houses images from a camera trap survey focused on reticulated giraffe (*Giraffa reticulata* – Fennessy et al., 2016) being conducted in two locations in Northern Kenya: Loisaba Conservancy (~23,000 ha) and Namunyak Community Conservancy (~405,000 ha). Loisaba Conservancy is characterized by a mix of savanna grasslands (Open Grasslands) and mixed acacia woodlands (*Acacia reficiens‐Acacia mellifera* Open/Sparse Woodlands) habitat (Unks, R. personal comment, 2016) whereas Namunyak Community Conservancy is composed of much more diverse vegetation classes ranging from various shrublands (*Grewia* spp, *Boscia coriacea, A. reficiens*), deciduous bushland, and dense evergreen forest (Chafota, 1998). At Loisaba Conservancy, 80 cameras were set out across a 207 km2 grid, offering systematic coverage of the entire study area. Within Namunyak Conservancy, 50 cameras were set out across a 207 km2 grid, covering only 5% of the entire area, due to the challenging terrain and limited mobility of the research team. All cameras deployed were Bushnell Trophy Cam HD cameras. Since February 2016, approximately 2 million images have been collected thus far. Cameras were set to collect one image per burst and were set to “auto” sensitivity, meaning the camera adjusted the trigger signal based its current operating temperature (Bushnell, 2014). On www.wildwatchkenya.org, each photograph was circulated to 10–20 volunteers (mean = 10), depending on agreement between volunteers, before it was retired. Since 2017, WWK has accrued over 16,700 volunteers and classified over 1.2 million images as of January 2020.

### 2.4 AmazonCam Tambopata

AmazonCam Tambopata classifies images from a camera trap survey being conducted within two protected areas in Peru: the Tambopata National Reserve (~275,000 ha) and the Bahuaja Sonene National Park (~1.1 million ha). The study's focus is to increase knowledge on Amazonian rainforest habitat and wildlife, with specific focus on quantifying jaguar populations in the area. 85 cameras have been set out across a 300 km2 grid, offering systematic coverage of 1.5% of the total area. Like WWK, all cameras deployed were Bushnell Trophy Cam HD cameras. Approximately 500,000 image sets were collected between July 2016 and December 2018. The cameras were set to capture three images per burst with “normal” sensitivity, an intermediate sensitivity level (Bushnell, 2014). On ACT, each camera trap photograph was circulated to 10–30 volunteers (mean = 13) before it was retired, depending on agreement among volunteers. ACT has accrued over 11,000 volunteers, who completed the classification of 10,000 images as of November 2019.

### 2.5 Data aggregation

A simple plurality algorithm was implemented on SS, WWK, and ACT, converting the multiple volunteer answers into one aggregated answer. This aggregated answer reports the species that had a majority of the votes for each photograph. For example, if a photograph had 15 total classifications from the 15 volunteers, where three classification were dik dik (*Madoqua kirkii*), five classifications were gazelle (*Gazella thomsonii* or *G. granti*), and seven were impala (*Aepyceros melampus*), the plurality algorithm would report the photograph to contain an impala (Swanson et al., 2016). This aggregated answer is hereafter referred to as the nonexpert answer (NEA).

### 2.6 Part I: Accuracy assessment

Photographs from each of the three projects were classified by experts into expert‐verified datasets, “Expert Answers” (EA). For each project, the NEA was compared to EA. The proportion of images where the NEA and the EA agreed is reported as the overall accuracy. For WWK and ACT, when NEA and the EA disagreed, the photograph was labeled as “false species” if the NEA falsely identified the species present, or “false empty” if the NEA falsely reported that there was no species in the image. The rates of overall accuracy across the three projects were compared using pairwise comparison of proportions. The rates of false empties and false species between WWK and ACT were also compared using a two proportion Z‐test. Images where the NEA reported more than one species present were excluded from the analysis.

For SS, a panel of five experts reviewed a randomly sampled set of 3,829 images to determine overall accuracy (Swanson et al., 2016). Each image was classified by one expert and was subsequently reviewed by a second expert if the image was flagged as difficult. The experts either had extensive formal training, passed qualification examinations, or had years of experience identifying African wildlife—see Swanson et al. (2016). For this set, false species and false empty levels were not analyzed because the study aimed to quantify overall accuracy and species level accuracy based on false positives and false negatives. It also should be noted that SS accuracy levels reported in this manuscript are all based on results obtained directly from Swanson et al. (2016).

For WWK, a panel of three experts reviewed a set of 127,669 images. We removed 84 images that the expert determined to be unidentifiable and limited analysis to the 24,039 images that contained only one type of species. Each photograph was classified by at least one expert with training and/or significant experience identifying African wildlife.

In the case of ACT, a panel of three experts reviewed a random subset of 4,040 images that contained only one type of species. Images of arboreal species were removed since the other datasets did not include arboreal species, leaving 2,598 images of terrestrial species for analysis. The experts either had significant experience identifying wildlife in the Peruvian Amazon or underwent extensive training.

### 2.7 Part II: Wildwatch Kenya extended classification set analysis

In order to look further into WWK’s lower rate of overall accuracy as compared to SS and ACT, and abundance of false empties compared to ACT, a separate analysis with a subset of 21,530 WWK images was conducted. This subset represented the images that had at least one citizen scientist classification of either a reticulated giraffe, a zebra (*Equus quagga* or *E. grevvi*), an elephant (*Loxodonta africana*), a gazelle, an impala, or a dik dik, and also had only one type of species present. These wildlife species were chosen because they had the highest frequency of appearance in WWK’s images, thus eliminating the possibility of inaccuracy due to rareness of the species as reported in Swanson et al. (2016). This methodology allowed scrutiny of images that potentially contain wildlife but were listed as empty by the aggregated NEA because not enough volunteers recognized that there was an animal in the photograph. For example, in an image containing a giraffe traveling in the far background, there was one citizen science classification of “giraffe,” but nine classifications of “empty.” In this case, the NEA would classify this photograph as empty because most citizen scientists did not notice the giraffe in the background. Utilizing this methodology, we hoped to recover as many wildlife photographs as possible that would have otherwise been weeded out by the plurality algorithm in order to quantify these incidences. This subset of photographs will hereafter be referred to as the Extended Classification Set.

An expert reviewed the images from the Extended Classification Set and determined the images that actually contained either a giraffe, a zebra, an elephant, a gazelle, an impala, or a dik dik. The aggregated NEA of those images were then compared to the EA to determine if the NEA agreed or disagreed with the EA. Similar to the above analysis, the proportion of photographs that agreed were represented as the overall accuracy rate for each of the six listed species, and photographs that disagreed were broken up by false empty and false species. The rate of overall accuracy was compared between each of the six species in the Extended Classification set using pairwise comparison of proportions. The same comparison and statistical analysis were performed for the rate of false species images and for the rate of false empty images between the six species.

### 2.8 Part III: Reason for false image classification

For images where the NEA and EA disagreed within ACT and the WWK Extended Classification Set, an expert conducted an additional review to determine the most likely reason for disagreement: distance (species was far in the background), night time (image was too dark to determine species), partial view (only a portion of the species was captured in the frame), close up (species was too close to the camera), hidden (vegetation or other obstacle impeding view of the species), or misidentification (species was confused with another species).

## 3 RESULTS

### 3.1 Part I: Overall accuracy assessment

When comparing the overall accuracy between WWK, SS, and ACT (images where the NEA and EA agreed/ total number of images), the NEA for WWK was the least accurate (83.4%; *n* = 20,050), followed by ACT (93.5%; *n* = 2,430), then SS (97.9%; *n* = 3,749) (Swanson et al., 2016). The proportions of false species images for WWK and ACT are 2% (*n* = 403) and 4% (*n* = 116), respectively. The proportions of false empties were WWK 15% (*n* = 3,586) and ACT 2% (*n* = 52). There was significant difference in overall accuracy between WWK, SS, and ACT (pairwise comparison of proportions; *p* < .0002; Ford, 2016; R Core Team, 2018). There was also significant difference in false empties and false species rates between WWK and ACT (two proportion *Z*‐test; *p* < .0002; *p* < .0002). WWK’s false empty images also constituted nearly 90% of its total error.

### 3.2 Part II: Wildwatch Kenya Extended Classification Set analysis

**FIGURE 2:**
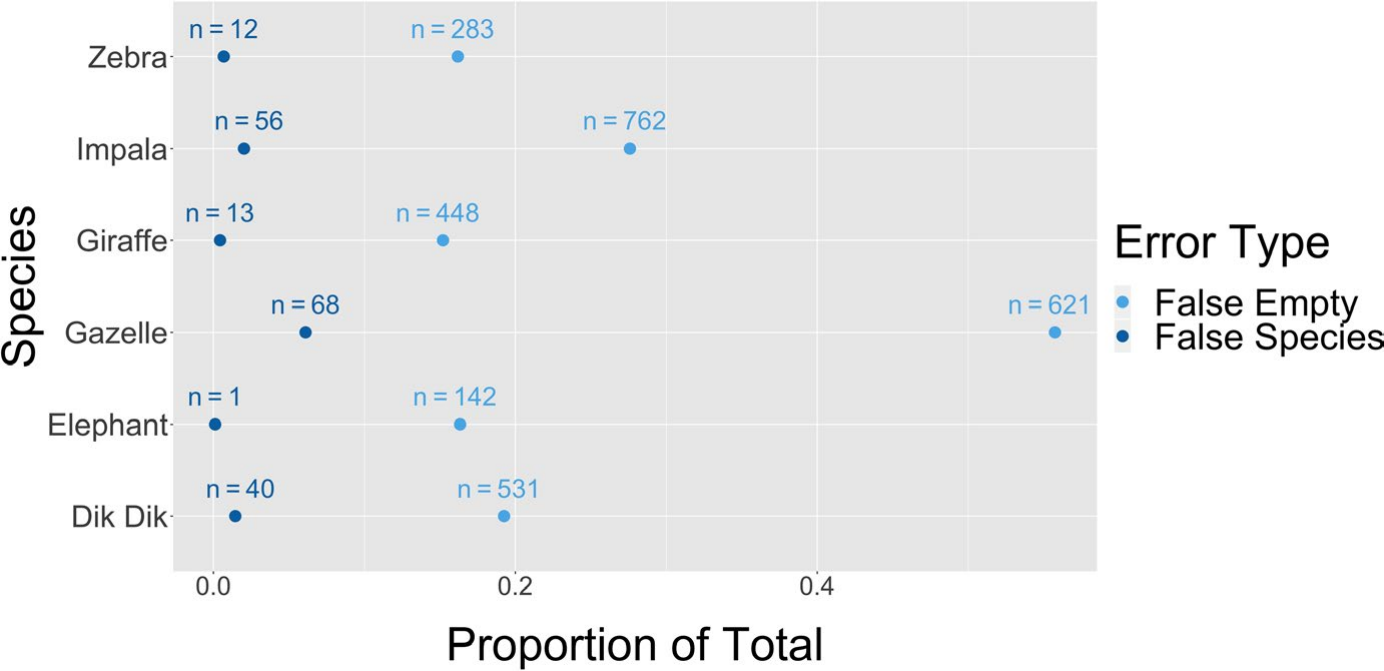
Comparison of the overall NEA false empty and false species images within WWK Extended Classification Set

**FIGURE 3:**
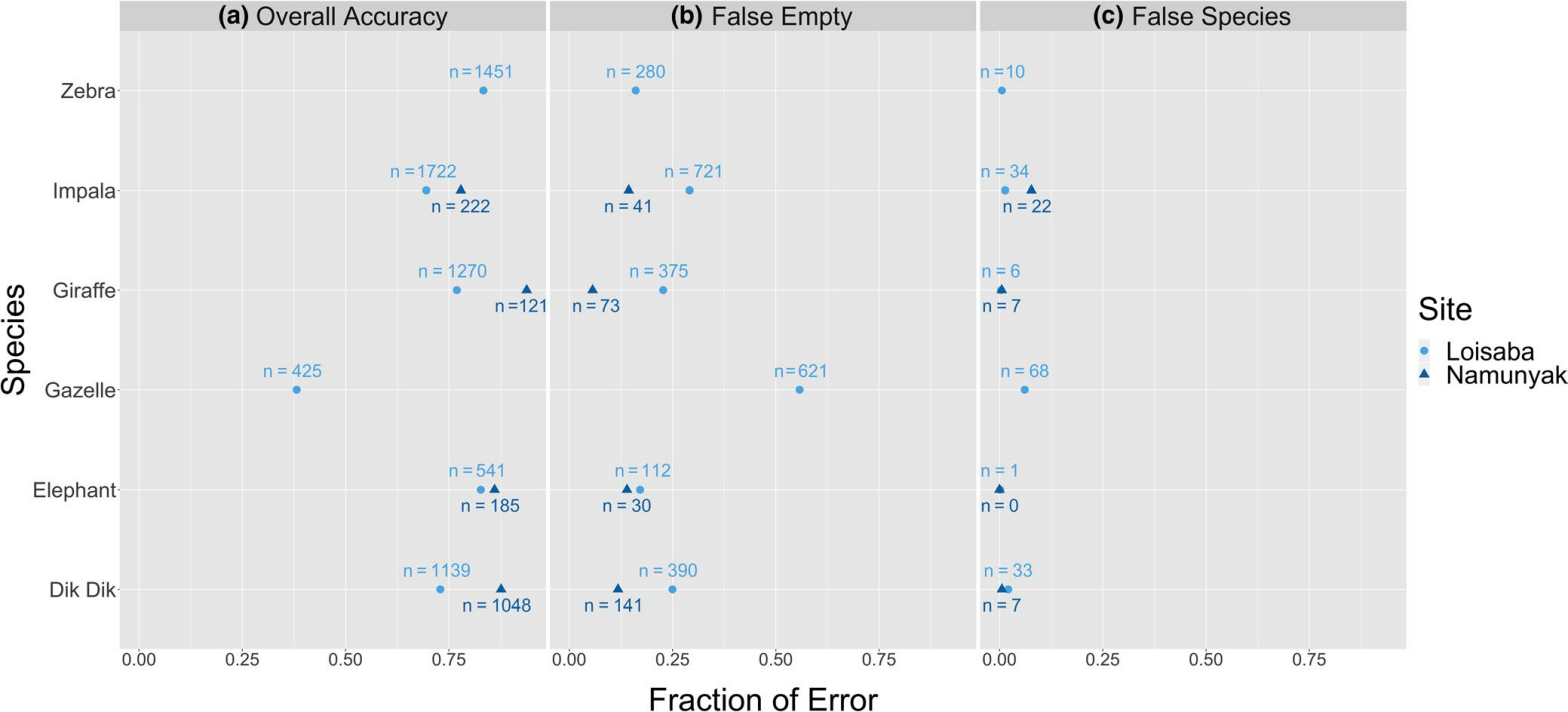
Comparison of the proportions of overall accuracy (a), false empty images (b) and false species images (c) between WWK Loisaba and WWK Namunyak sites for each of the six species analyzed to differentiate the effect of different habitat types. Namunyak's values for zebra removed due to the low sample size

The expert reviewed the Extended Classification Set and determined that 12,197 of the 21,530 images actually contained images of either a giraffe, a zebra, an elephant, a gazelle, an impala, or a dik dik. The overall accuracy of these 12,197 images was 75.7%, representing a 7.7% accuracy decrease from Part I WWK analysis. However, the rates of false species error are very low for each species (≤6%; Figure 2). This suggests that when the citizen scientists recognized that there was an animal in the image, they frequently classified the species correctly. Using pairwise comparison of proportions, we determined that the proportion of false empty images was significantly higher than the proportion of false species images (*p* < .0002) for every species analyzed, meaning there were many images where the NEA reported a blank image, but the expert reported a species. For the photographs that the expert determined to have gazelle, the citizen scientists labeled over half (55%) as empty. To examine this discrepancy further, WWK’s two different sampling sites were analyzed separately (Figure 3). Loisaba had a significantly higher proportion of false empties (27%) compared with Namunyak (9.6%) (*p* < .0002).

### 3.3 Part III: Reason for false image classification

**FIGURE 4:**
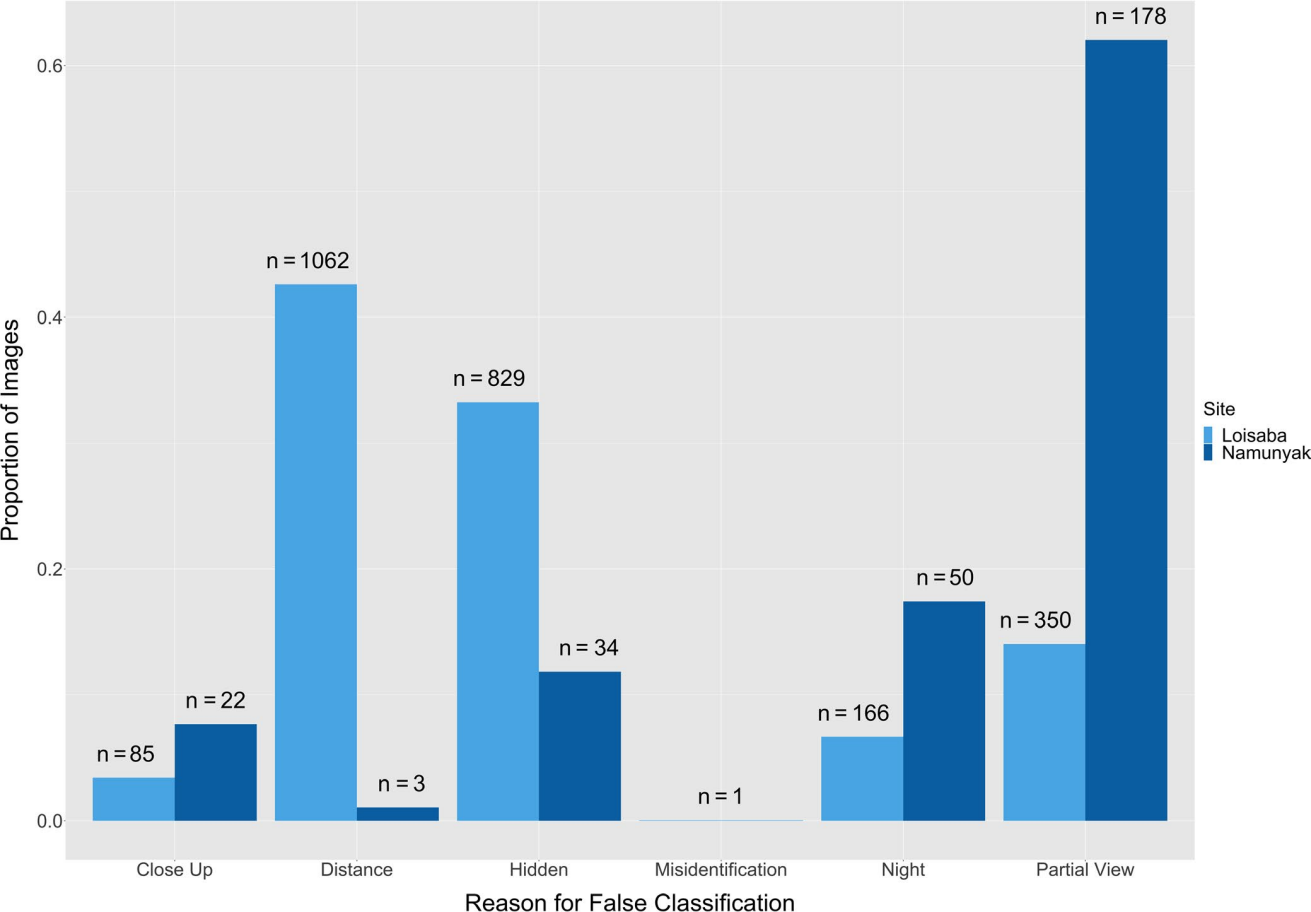
“False empty” proportion of WWK Extended Classification Set images for WWK Loisaba and WWK Namunyak sites. These “false empty” categories include: close up (species was too close to the camera), distance (species was far in the background of the image), hidden (vegetation or other obstacle impeding view of the species), misidentification (species was confused with another species), night (image was too dark to determine species), or partial view (only a portion of the species was captured in the frame)

The false species and false empty images were reviewed by the expert post hoc to determine the most likely reason that the photograph was incorrectly classified. In Loisaba, nearly half of the false species (45%) and false empty (42%) images were because the animal was far off in the distance (Figure 4). For Namunyak, a majority of the false empty (61%), and the most frequent reason for false species (38%), were due to a partial view of the animal, mostly from the individual entering or exiting the frame (Figure 4). In comparison, none of the error within ACT was due to distance, as the depth and width of view were limited by the dense vegetation.

## 4 DISCUSSION

Of the three studies, WWK had the lowest accuracy levels, with the error mainly due to the high number of false empty images (15%). This suggests that WWK volunteers were simply not seeing animals in the frame, and falsely classifying the photograph to be empty. Comparatively, ACT had a much lower rate of false empties (2%). If WWK were able to increase species detectability, and thus reduce the number of false empty images to this same rate of 2%, WWK’s overall accuracy would increase to 96.3%. Comparing the differences between these projects (Table 2), we suggest that WWK error, and the resulting discrepancy in accuracy, can be attributed to three factors: the number of images taken per trigger, the camera sensitivity, and the habitat types.

**TABLE 2:** Camera trap sensitivity setting, number of images that were captured per trigger event, camera trap sensitivity setting, and habitat types of the three citizen science projects

Project	Number of images per trigger	Camera sensitivity	Habitat type
Snapshot Serengeti	1–3 (majority 3)	Low	Savanna Grasslands and Savanna Woodlands
Wildwatch Kenya	1	Auto	Loisaba: Savanna Grasslands Namunyak: Savanna Woodlands
AmazonCam Tambopata	3	Medium	Rainforest

Overall accuracy was increased when cameras were set to take three images per trigger rather than one single image. Small species (e.g., small rodents) or species that appear small in an image due to the distance from the camera are most easily detected by observers based on pixels changing in consecutive images of the same scene. In SS and ACT, the three consecutive photographs per trigger instance were presented in Zooniverse as a slideshow, showing the volunteers small changes in the frames from one photograph to the next while for WWK a single image was presented. Because the images on Zooniverse are presented to the volunteers in random order, change‐detection from one image to the next was not possible. In contrast, the experts reviewing the WWK photographs viewed images in order of progression and could detect the animals due to changes in pixels from one image to the next.

We further predict that sequences of three photographs will reduce misidentifications due to “partial view” and “hidden” because the animal will likely come into full view within the three‐photograph sequence, rather than a single frame only showing a small portion of the body (Rovero, Zimmermann, Berzi, & Meek, 2013). Because “distance,” “hidden,” and “partial view” were the most frequently cited reason for false empty error within WWK, using three photographs would have significantly increased WWK’s overall accuracy. Although more than three images per trigger may further increase accuracy, more images also add time for both citizen scientists and experts when classifying images. Thus, we suggest that the use of three consecutive photographs per trigger instance increases accuracy of citizen science classifications of wildlife images.

Further, because there was not an “I don't know” option within WWK, it is possible that some false empties from “partial view” resulted from volunteers opting for an “empty” classification rather than taking a guess of what the species is (Swanson et al., 2016). Including an “I don't know” option could decrease the number of false empties because experts would be able to go through the images marked as unsure and determine the correct classification, rather than having these images marked as “empty” by the plurality algorithm. However, it should be noted that having an “I don't know” option may also discourage citizen scientists from taking their best guess (Swanson et al., 2015). It also should be noted that according to findings from Swanson et al. (2015), image classification accuracy increases with increasing citizen science classification up to 10 classifications, then levels off. Thus, because the images in all three projects had at least 10 classifications, the differing number of classifications on each photograph between the three projects should not have impacted the rate accuracy.

The WWK images from Loisaba Conservancy had a higher rate of false empties compared with Namunyak Conservancy. The camera trap methodology was the same at both sites, apart from the habitat type (Table 3). Thus, we can attribute this increased rate of inaccuracy to the open, grassy habitat in Loisaba (Figures 3 and 4). In open habitat, images triggered by heat or vegetation capture animals in the background of the frame at distances that would not otherwise trigger the camera (Koivuniemi, Auttila, Niemi, Levänen, & Kunnasranta, 2016; Rovero et al., 2013; Wearn & Glover‐Kapfer, 2019). WWK volunteers often missed the classification of such animals in the distance because there was only one photograph per image set, rather than three, causing an increased rate of false empty classification due to “distance.” This rate of misfires and the subsequent rate of false empty images were not seen within ACT rainforest habitat, where dense vegetation blocks wind currents and keeps the foliage still. ACT cameras only misfired 17.8% of the time, while WWK camera misfired 81% of the time, and SS cameras misfiring at a lesser rate of 74% (Swanson et al., 2015). We recognize that how a species appears in the field of view cannot be controlled in a natural setting. However, given these findings, we recommend that 3 consecutive images be used in order to detect small changes in the background of images, thus reducing the likelihood of misclassification.

Camera trap sensitivity settings also affect accuracy rates. When camera sensitivity is set to “high,” camera misfiring due to moving vegetation or heat is increased. In “low” sensitivity, smaller or rapidly moving animals may not trigger the camera. Standard camera‐trapping protocols recommend a “high” sensitivity setting for warm climates (Meek, Fleming, & Ballard, 2012; Rovero & Zimmermann, 2016). However, based on the WWK results, the high sensitivity setting caused the camera to misfire frequently. Of the 127,669 WWK images reviewed by the expert, only 19% (*n* = 24,039) contained species, and 81% (*n* = 103,630) of the photographs were assumed to be misfires. As such, we recommend that the cameras be tested on a number of different sensitivity settings before selecting a final setting for the study site, with consideration of environmental context, the species of interest, and the method of image classification. In this study, we were not able to quantify if a lower sensitivity setting would have missed species images for the three projects (Table 3).

**TABLE 3:** Comparison of accuracy and inaccuracy rates between the each Zooniverse project (Wildwatch Kenya, Snapshot Serengeti, and AmazonCam) showing where the expert answer did not agree with the aggregated volunteer answer (“Number Incorrect”), and the reason for the citizen science inaccuracy. (See Swanson et al., 2016 for details Snapshot Serengeti accuracy rates)

Site	Total expert verified images analyzed	Number incorrect	Number false species	Number false empty	Prop correct	Prop incorrect	Prop false species	Prop false empty
Wildwatch Kenya	24,039	3,989	403	3,586	0.83	0.17	0.02	0.15
WWK extended classification	12,197	2,974	190	2,787	0.76	0.24	0.02	0.23
Loisaba	9,199	2,649	152	2,499	0.71	0.29	0.02	0.27
Namunyak	2,998	325	38	288	0.89	0.11	0.01	0.10
AmazonCam	2,598	168	116	52	0.94	0.06	0.04	0.02
Snapshot Serengeti	3,829	79			0.98	0.02		

A recent focus of camera trap literature has been on automatic classification through machine learning. Deep convolutional neural networks are trained to automatically and accurately identify, count, and describe species in camera trap images (Norouzzadeh et al., 2018). However, a drawback of automatic classification is the need for a large set of preclassified images for baseline training data (Willi et al., 2019). Crowdsourced citizen science can quickly produce the training data for deep learning models, making its use still relevant. In addition, combining automation and crowdsourcing may improve automatic classification accuracy, and significantly decrease time commitment of volunteers (Willi et al., 2019). Automated classification integration could be a next development for crowdsourcing platforms like Zooniverse, where pretrained models are added into data‐processing pipelines and the resulting predictions are combined with citizen scientist classification predictions. For example, WWK has already begun this cross‐method analysis by utilizing automated classification models from Willi et al. (2019) to preprocess out empty images with confidence >0.80, thus only uploading images for citizen science classification with high confidence of containing an animal, or a low confidence that it is empty. This significantly reduces the time commitment and fatigue for volunteers and increases volunteer engagement by removing empty images.

Overall, WWK consensus answers had high species classification accuracy. However, there was a discrepancy in the overall accuracy between WWK and both SS and ACT because WWK’s aggregated NEA often reported the photograph as empty, when in fact it contained a species. Thus, WWK’s aggregated NEA currently underestimates the number of species images captured. The evidence presented here shows that WWK’s error is due to single photograph per trigger instance versus the three photographs per trigger instance, camera misfires caused by Loisaba's open, grassy habitat, which captured animals too far in the distance for citizen scientists to see, and to WWK’s cameras set on auto sensitivity, which often defaulted to “high” due to Northern Kenya's warm climate. Our analyses provide a foundation from which to develop standardized, evidence‐based best practices for camera trap‐based studies that engage citizen scientists. Implementation of our findings should result in increases in species detectability and image classification accuracy, which are both critical for meeting research goals. Optimizing citizen science accuracy and validating the resulting data will increase the usability of nonexpert data for applied science. Once validated, tapping into volunteer participation can exponentially increase the speed at which scientific data are collected and processed at little to no cost, and have the potential to revolutionize the way we think about science.

## CONFLICT OF INTEREST

We have no conflicts of interest to report for this manuscript.

## AUTHOR CONTRIBUTION

**Nicole Egna:** Conceptualization (equal); Data curation (equal); Formal analysis (lead); Methodology (equal); Visualization (lead); Writing‐original draft (lead); Writing‐review & editing (equal). **David O'Connor:** Conceptualization (equal); Funding acquisition (lead); Methodology (equal); Project administration (lead); Resources (lead); Supervision (lead); Validation (supporting); Writing‐review & editing (lead). **Jenna Stacy‐Dawes:** Conceptualization (equal); Data curation (lead); Funding acquisition (equal); Methodology (equal); Project administration (lead); Supervision (lead); Writing‐review & editing (supporting). **Mathias W. Tobler:** Methodology (supporting); Validation (supporting); Writing‐review & editing (lead). **Nicholas Pilfold:** Data curation (supporting); Project administration (supporting); Writing‐review & editing (supporting). **Kristin Neilsen:** Data curation (supporting); Software (supporting); Writing‐review & editing (supporting). **Brooke Simmons:** Data curation (supporting); Resources (supporting); Software (lead); Validation (supporting); Writing‐review & editing (supporting). **Elizabeth Oneita Davis:** Investigation (supporting); Methodology (supporting); Project administration (supporting); Writing‐review & editing (supporting). **Mark Bowler:** Data curation (supporting); Project administration (supporting); Writing‐review & editing (supporting). **Julian Fennessy:** Writing‐review & editing (equal). **Jenny Anne Glikman:** Funding acquisition (equal); Project administration (equal); Writing‐review & editing (supporting). **Lexson Larpei:** Data curation (supporting); Writing‐review & editing (supporting). **Jesus Lekalgitele:** Data curation (supporting); Writing‐review & editing (supporting). **Ruth Lekupanai:** Data curation (supporting); Writing‐review & editing (supporting). **Johnson Lekushan:** Data curation (supporting); Writing‐review & editing (supporting). **Lekuran Lemingani:** Data curation (supporting); Writing‐review & editing (supporting). **Joseph Lemirgishan:** Data curation (supporting); Writing‐review & editing (supporting). **Daniel Lenaipa:** Data curation (supporting); Project administration (supporting); Writing‐review & editing (supporting). **Jonathan Lenyakopiro:** Data curation (supporting); Project administration (supporting); Writing‐review & editing (supporting). **Ranis Lenalakiti Lesipiti:** Data curation (supporting); Writing‐review & editing (supporting). **Masenge Lororua:** Data curation (supporting); Writing‐review & editing (supporting). **Arthur Muneza:** Writing‐review & editing (equal). **Sebastian Rabhayo:** Data curation (supporting); Writing‐review & editing (supporting). **Symon Masiaine Ole Ranah:** Data curation (supporting); Funding acquisition (supporting); Project administration (supporting); Writing‐review & editing (supporting). **Kirstie Ruppert:** Funding acquisition (equal); Project administration (equal); Writing‐review & editing (supporting). **Megan Owen:** Funding acquisition (supporting); Project administration (lead); Supervision (lead); Writing‐review & editing (equal).

## Supporting information

Supplementary Material

## Data Availability

The expert‐verified datasets for AmazonCam Tambopata and Wildwatch Kenya, including the Extended Classification Set, are made available on Dryad (https://doi.org/10.5061/dryad.n02v6wwv9).
