# Supplementary material for: Camera settings and biome influence the accuracy of citizen science approaches to camera trap image classification
Source: Ecol Evol. 2020 Oct 6;10(21):11954–65. doi: 10.1002/ece3.6722 (PMC7663993; doi:10.1002/ece3.6722)
Supplement: Supplementary file 1 — Supplementary Material [file ECE3-10-11954-s001.docx]

**Appendix S2** The number of images correct and incorrect, as well as the associated rate of error for the Wildwatch Kenya Extended Classification Set (the group of photos where at least one volunteer classified a dik dik, elephant, gazelle, giraffe, impala, or zebra). ‘Total’ is the total number of photos for that species in the specified citizen science project, ‘n’ is the number of photos incorrect (either false empty or false species), ‘Frequency’ is the proportion of error, and ‘s’ is the difference of the values of ‘total’ and ‘n’, i.e. number of photos correct.

| Variable | total | n | Frequency | s |
| --- | --- | --- | --- | --- |
| Dik Dik  Loisaba  False Empty  False Species  Namunyak  False Empty  False Species | 1562  1196 | 390  33  141  7 | 0.249679898  0.021126761  0.117892977  0.005852843 | 1172  1529  1055  1189 |
| Elephant  Loisaba  False Empty  False Species  Namunyak  False Empty  False Species | 654  215 | 112  1  30  0 | 0.171253823  0.001529052  0.139534884  0 | 542  653  189  215 |
| Gazelle  Loisaba  False Empty  False Species  Namunyak  False Empty  False Species | 1114  0 | 621  68  0  0 | 0.557450628  0.061041293  NA  NA | 493  1046  0  0 |
| Giraffe  Loisaba  False Empty  False Species  Namunyak  False Empty  False Species | 1651  1295 | 375  6  73  7 | 0.22713507  0.003634161  0.056370656  0.005405405 | 1276  1645  1222  1288 |
| Impala  Loisaba  False Empty  False Species  Namunyak  False Empty  False Species | 2477  285 | 721  34  41  22 | 0.291077917  0.013726282  0.143859649  0.077192982 | 1756  2443  244  263 |
| Zebra  Loisaba  False Empty  False Species  Namunyak  False Empty  False Species | 1741  7 | 280  10  3  2 | 0.160827111  0.005743825  0.428571429  0.285714286 | 1461  1731  4  5 |
